# Bilateral eyelid erythema and oedema accompanied by systemic lupus erythematosus and syndrome of inappropriate antidiuretic hormone secretion

**DOI:** 10.1093/rap/rkag040

**Published:** 2026-03-31

**Authors:** Kohei Goda, Takashi Nawata, Mako Yonezawa, Natsumi Nishimura, Masafumi Fujinaka, Masaki Shibuya, Hiroshi Nakamura, Motoaki Sano

**Affiliations:** Department of Medicine and Clinical Science, Yamaguchi University Graduate School of Medicine, Ube, Japan; Department of Medicine and Clinical Science, Yamaguchi University Graduate School of Medicine, Ube, Japan; Department of Medicine and Clinical Science, Yamaguchi University Graduate School of Medicine, Ube, Japan; Department of Medicine and Clinical Science, Yamaguchi University Graduate School of Medicine, Ube, Japan; Department of Medicine and Clinical Science, Yamaguchi University Graduate School of Medicine, Ube, Japan; Department of Medicine and Clinical Science, Yamaguchi University Graduate School of Medicine, Ube, Japan; Department of Medicine and Clinical Science, Yamaguchi University Graduate School of Medicine, Ube, Japan; Department of Medicine and Clinical Science, Yamaguchi University Graduate School of Medicine, Ube, Japan

A 44-year-old Japanese man with bilateral eyelid erythema and oedema was admitted to our hospital (Fig. 1A and B). Physical examination did not detect any other abnormal findings, such as butterfly erythema, polyarthritis, muscle weakness or leg oedema. Laboratory findings suggested systemic lupus erythematosus (SLE) (Supplementary Table S1). Computed tomography revealed pericardial fluid, pleural effusion and ascites. Echocardiography suggested euvolemia. Hypoalbuminemia (2.9 g/dl) and proteinuria (0.49 g/24 h) were also observed. Renal biopsy suggested Class II lupus nephritis. He was diagnosed with SLE.

Furthermore, hyponatraemia (119 mmol/l) was observed. The urine sodium concentration was 27 mmol/l. Elevated serum antidiuretic hormone levels (5.7 pg/ml), urine osmolality (108 mOsm/kg) and low plasma osmolality (251 mOsm/kg) indicated the syndrome of inappropriate secretion of antidiuretic hormone (SIADH). Drug-induced hyponatraemia was excluded. After consultation with an endocrinologist, he was diagnosed with SIADH.

He was treated with prednisolone (initial dose: 60 mg/day) and mycophenolate mofetil, which improved eyelid erythema but not oedema (Fig. 1C). In addition, tolvaptan administration effectively alleviated eyelid oedema (Fig. 1D). Serum sodium concentration increased to 135 mmol/l.

**Figure 1:**
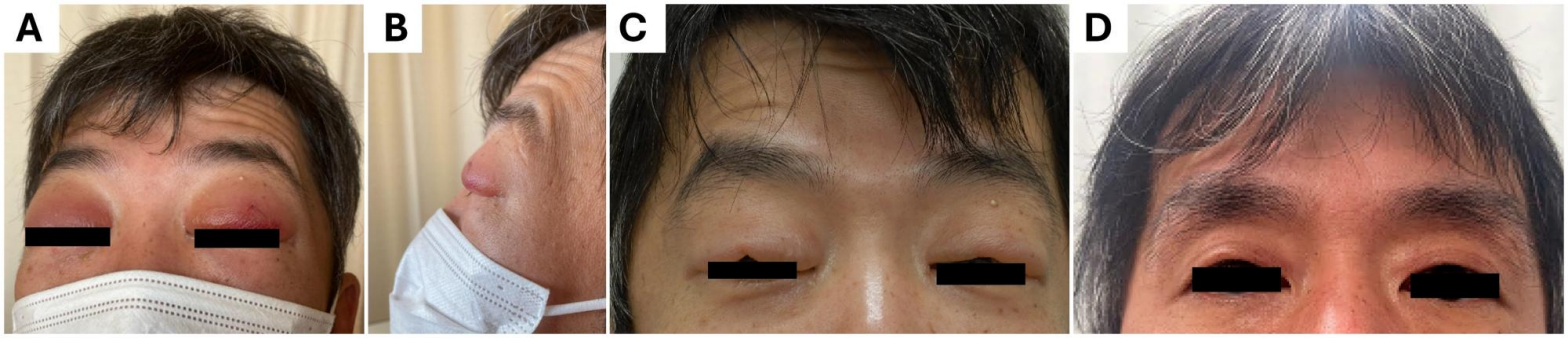
Imaging findings of the patient. (A and B) Bilateral eyelid erythema and oedema were identified on admission. (C) Eyelid appearance after treatment with prednisolone for 21 days (60 mg/day for 14 days, followed by 30 mg/day for 7 days; prednisolone was rapidly reduced after the renal biopsy results were reported) and mycophenolate mofetil for 4 days (500 mg/day). The very short duration of mycophenolate mofetil therapy suggests that the improvement in eyelid erythema was most likely attributable to corticosteroid treatment. (D) Eyelid findings after hospital discharge

The concurrent presence of eyelid erythema and oedema in SLE is rare [1, 2]. We hypothesised that the oedema was driven by ADH-mediated water retention, superimposed on low oncotic pressure resulting from protein loss due to SLE. Clinicians should consider SIADH in patients with SLE who present with unexplained physical findings and hyponatraemia.

## Supplementary Material

rkag040_Supplementary_Data

## Data Availability

The authors declare that all the relevant data supporting the findings of this case report are available within the article.
