# Supplementary material for: Bilateral eyelid erythema and oedema accompanied by systemic lupus erythematosus and syndrome of inappropriate antidiuretic hormone secretion
Source: Rheumatol Adv Pract. 2026 Mar 31;10(2):rkag040. doi: 10.1093/rap/rkag040 (PMC13064973; doi:10.1093/rap/rkag040)
Supplement: rkag040_Supplementary_Data [file rkag040_supplementary_data.docx]

**Supplementary Table S1. Clinical data at the time of diagnosis of systemic lupus erythematosus**

Based on the above findings, alternative causes of bilateral eyelid erythema/oedema, such as IgG4-related disease, dermatomyositis, and hereditary angioedema, were excluded. In contrast, the patient fulfilled the 2019 ACR/EULAR classification criteria for systemic lupus erythematosus (domains met: antinuclear antibodies, leukopenia, positivity for anti-dsDNA antibodies and anti-Sm antibodies, hypocomplementemia, pleural and pericardial effusion, renal biopsy findings).

WBC: White blood cell, AST: Aspartate Aminotransferase, ALT: Alanine Aminotransferase, LDH: Lactate Dehydrogenase, BUN: Blood Urea Nitrogen, CRP: C-Reactive Protein, Na: Sodium, K: Potassium, Cl: Chlorine, IgG: immunoglobulin G, TSH: Thyroid-Stimulating Hormone, BNP: Brain Natriuretic Peptide.
